# Supplementary material for: Data from the German TwinLife Study: Genetic and Social Origins of Educational Predictors, Processes, and Outcomes
Source: J Open Psychol Data. 2023 Mar 21;11:4. doi: 10.5334/jopd.78 (PMC12270247; doi:10.5334/jopd.78)
Supplement: Appendix. — Figures A1 to A4. [file jopd-11-78-s1.pdf]

Appendix

Figure A1

Distribution of zygosity across data collections

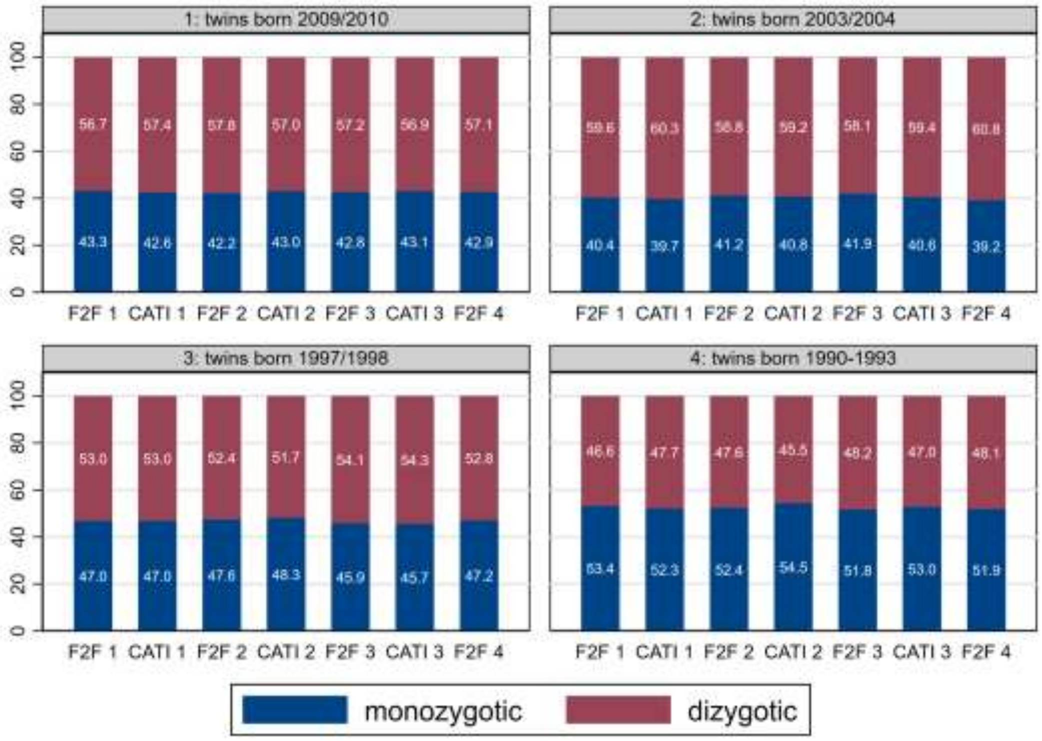

Note. Calculations for figures are based on TwinLife Data file Version 6.1.0, https://doi.org/10.4232/1.13987 (F2F1 to F2F3) and preliminary data for recently collected data (CATI3, F2F4).

Figure A2

Distribution of twins' sex across data collections

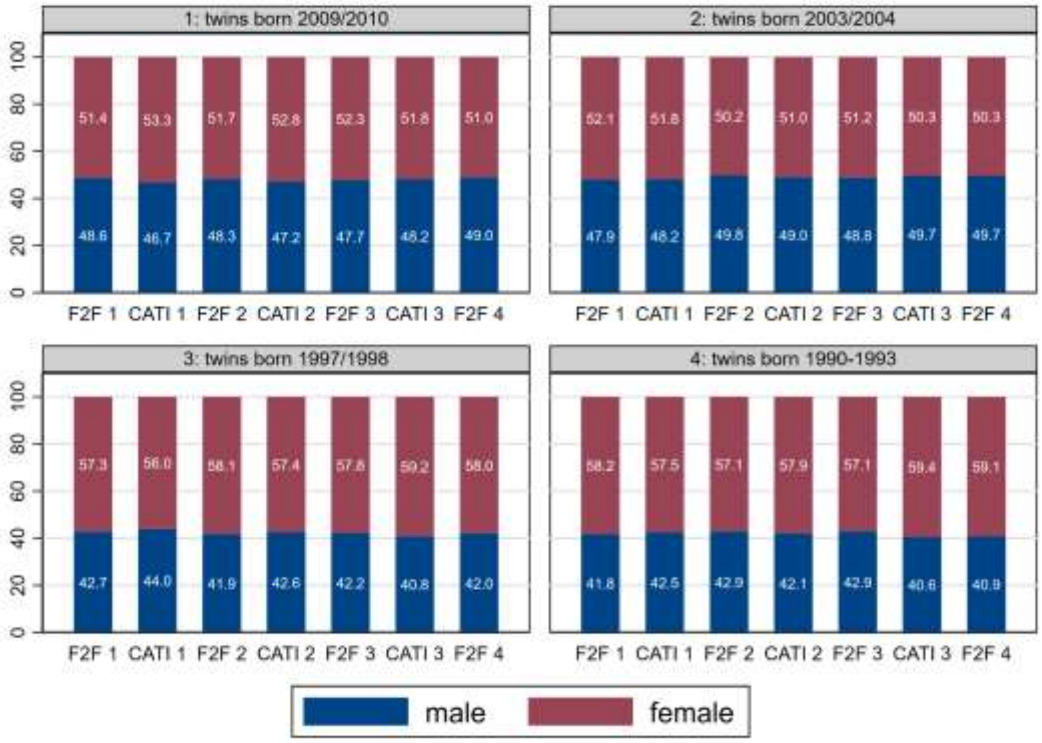

Note. Calculations for figures are based on TwinLife Data file Version 6.1.0, https://doi.org/10.4232/1.13987 (F2F1 to F2F3) and preliminary data for recently collected data (CATI3, F2F4).

Figure A3

Distribution of twins' migrations background across data collections

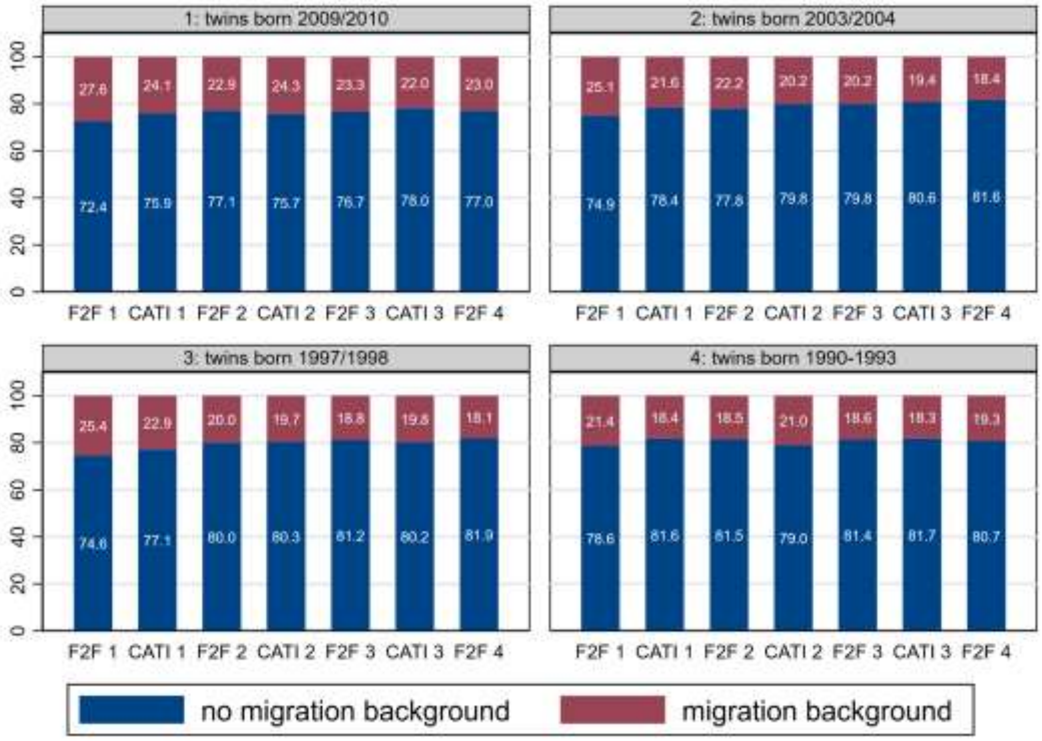

Note. Calculations for figures are based on TwinLife Data file Version 6.1.0, https://doi.org/10.4232/1.13987 (F2F1 to F2F3) and preliminary data for recently collected data (CATI3, F2F4). Migration background is assigned if either the twins were born abroad (first generation) or at least one parent was born abroad (second generation). Missing self-reports of the parents are filled in with the twins' proxy information on the parents' place of birth. In the case of completely missing information, it is assumed that there is no migration background.

Figure A4

Distribution of mother's education level across data collections

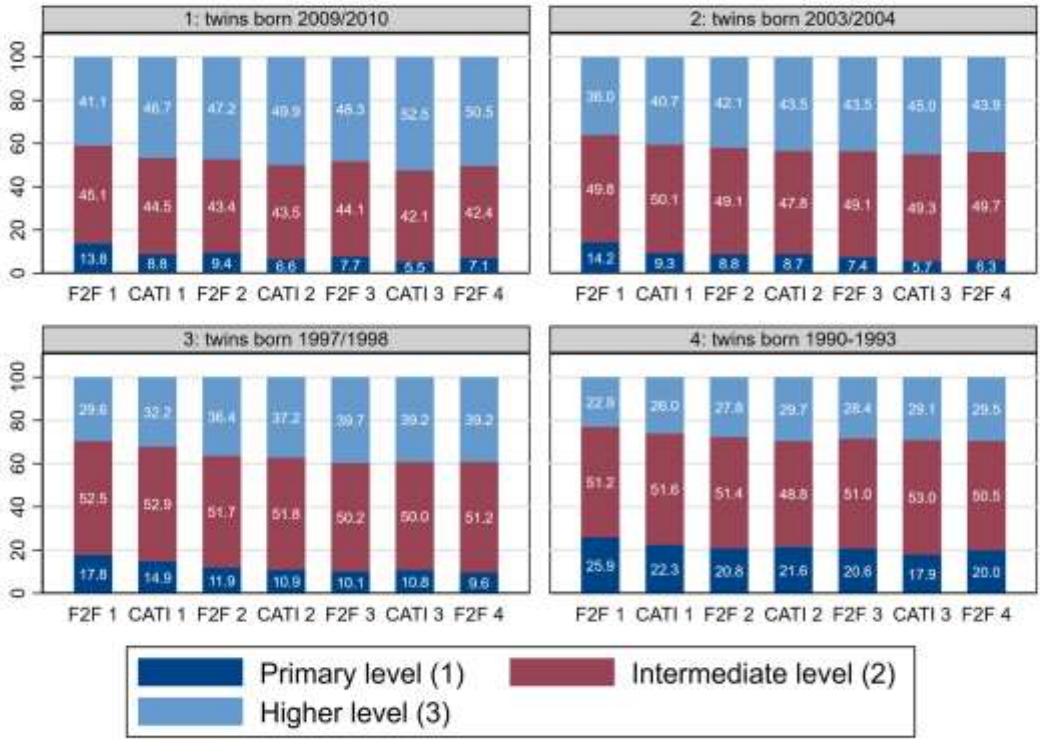

Note. Calculations for figures are based on TwinLife Data file Version 6.1.0, https://doi.org/10.4232/1.13987 (F2F1 to F2F3) and preliminary data for recently collected data (CATI3, F2F4). Mother’s education at F2F1 is based on CASMIN; missing information has been filled with father's education.

Publications related to education using TwinLife data

Baier, T. (2019). Does sibling and twin similarity in cognitive ability differ by parents' education?. ZfF–Zeitsschrift für Familienforschung/Journal of Family Research, 31(1), 58-82. https://doi.org/10.3224/zff.v31i1.04

Baier, T. & Lang, V. (2019). The Social Stratification of Environmental and Genetic Influences on Education: New Evidence Using a Register-Based Twin Sample. Sociological Science, 6, 143-171. https://doi.org/10.15195/v6.a6

Baier, T. & Van Winkle, Z. (2020). Does Parental Separation Lower Genetic Influences on Children's School Performance?. Journal of Marriage and Family. https://doi.org/10.1111/jomf.12730

Dings, A. & Spinath, F. M. (2021). Motivational and personality variables distinguish academic underachievers from high achievers, low achievers, and overachievers. Social Psychology of Education, 24, 1461–1485. https://doi.org/10.1007/s11218-021-09659-2

Eifler, E. F., Starr A., & Riemann, R. (2019). The genetic and environmental effects on school grades in late childhood and adolescence. PLoS ONE, 14(12). https://doi.org/10.1371/journal.pone.0225946

Eifler, E.F. & Riemann, R. (2022). The aetiology of educational attainment: A nuclear twin family study into the genetic and environmental influences on school leaving certificates. British Journal of Educational Psychology, 92(3), 881-892. https://doi.org/10.1111/bjep.12478

Gil-Hernández, C. J. (2019). Do Well-off Families Compensate for Low Cognitive Ability? Evidence on Social Inequality in Early Schooling from a Twin Study. Sociology of Education, 92(2), 150-175. https://doi.org/10.1177/0038040719830698

Gottschling, J., Hahn, E., Beam, C. R., Spinath, F. M., Carroll, S., & Turkheimer, E. (2019). Socioeconomic status amplifies genetic effects in middle childhood in a large German twin sample. Intelligence, 72, 20-27. https://doi.org/10.1016/j.intell.2018.11.006

Grätz, M., Lang, V., & Diewald, M. (2022). The effects of parenting on early

adolescents' noncognitive skills: Evidence from a sample of twins in Germany. Acta Sociologica, 65(4), 398-419. https://doi.org/10.1177/00016993211051958

Hahn, E., Gottschling, J., Bleidorn, W., Kandler, C., Spengler, M., Kornadt, A. E., Schulz, W., Schunck, R., Baier, T., Krell, K., Lang, V., Lenau, F., Peters, A.-L., Diewald, M., Riemann, R., & Spinath, F. M. (2016). What Drives the Development of Social Inequality Over the Life Course? The German TwinLife Study. Twin Research and Human Genetics, 19(6), 659-672. https://doi.org/10.1017/thg.2016.76

Mönkediek, B. (2020). Trait-specific testing of the equal environment assumption: The case of school grades and upper secondary school attendance. Journal of Family Research, 1-33. https://doi.org/10.20377/jfr-381

Mönkediek, B. (2022). How variants of tracking affect the role of genes and environment in explaining child attendance at upper secondary school. Research in Social Stratification and Mobility, 81, 100714. https://doi.org/10.1016/j.ssresearch.2021.102625

Mönkediek, B. & Diewald, M. (2021). Do academic ability and social background influence each other in shaping educational attainment? The case of the transition to secondary education in Germany. Social Science Research.

Paulus, L., Spinath, F. M., & Hahn, E. (2021). How do educational inequalities develop? The role of socioeconomic status, cognitive ability, home environment, and self-efficacy along the educational path. Intelligence, 86, 101528. https://doi.org/10.1016/j.intell.2021.101528

Schulz, W., Schunck, R., Diewald, M., & Johnson, W. (2017). Pathways of Intergenerational Transmission of Advantages during Adolescence: Social Background, Cognitive Ability, and Educational Attainment. Journal of Youth and Adolescence, 46(10), 2194-2214. https://doi.org/10.1007/s10964-017-0718-0

Spinath, F. M. & Bleidorn, W. (2017). The New Look of Behavioral Genetics in Social Inequality: Gene- Environment Interplay and Life Chances. Journal of Personality, 85(1), 5-9. https://doi.org/10.1111/jopy.12268

Starr, A. & Riemann, R. (2022). Chasing Environmental Influences on School Grades

in Childhood and Adolescence. Contemporary Educational Psychology, 69, 102043. (published online ahead of print) https://doi.org/10.1016/j.cedpsych.2022.102043

Starr, A., & Riemann, R. (2022). Common genetic and environmental effects on cognitive ability, conscientiousness, self-perceived abilities, and school performance. Intelligence, 93, 101664. https://doi.org/10.1016/j.intell.2022.101664

Stienstra, K., Maas, I., Knigge, A., & Schulz, W. (2021). Resource Compensation or Multiplication? The Interplay between Cognitive Ability and Social Origin in Explaining Educational Attainment. European Sociological Review, 37(2), 186–200. https://doi.org/10.1093/esr/jcaa054
